# Theoretical Calculations and Experiments on the Thermal Properties of Fluorinated Graphene and Its Effects on the Thermal Decomposition of Nitrate Esters

**DOI:** 10.3390/nano12040621

**Published:** 2022-02-13

**Authors:** Saiqin Meng, Xiaolong Fu, Liping Jiang, La Shi, Xu Wang, Xiangyang Liu, Jiangning Wang

**Affiliations:** 1 Xi’an Modern Chemistry Research Institute, Xi’an 710065, China; msq204@163.com (S.M.); jiangliping204@163.com (L.J.); shla_204@163.com (L.S.); 2 State Key Laboratory of Polymer Material and Engineering, College of Polymer Science and Engineering, Sichuan University, Chengdu 610065, China; wangxu@scu.edu.cn (X.W.); lxy6912@sina.com (X.L.)

**Keywords:** two dimensional materials, energetic materials, fluorinated graphene, nitrate esters, thermal decomposition

## Abstract

Fluorinated graphene contains F atoms with high levels of chemical activity, and the application of fluorinated graphene in energetic materials may greatly contribute to the progress of combustion reactions. However, there is a lack of research on the thermal properties of fluorinated graphene and its application on nitrate esters. In this paper, theoretical calculations and experiments were used to study the thermal properties of fluorinated graphene and its application on nitrate esters. The anaerobicity and poor thermal stability of fluorinated graphene were proved by ab initio molecular dynamics (AIMD) calculations and TG-DSC experiments. The ester weakening effect of fluorinated graphene on nitroglycerin was determined via wavefunction analysis, with the greater the fluorination degree, the stronger the ester weakening effect. The existence of fluorinated graphene can significantly increase the heat dissipation of the composites, which was concluded by TG-DSC experiments and TG-DSC-MS-FTIR. The research in this article provides an important reference for the application of fluorinated graphene in energetic materials.

## 1. Introduction

Fluorinated graphene (FG) has been known to be a typical derivative of graphene since it was first prepared in 2010 [1,2]. It has been used in many fields for its exceptional properties, such as its tunable bandgap [3], adjustable magnetic properties [4] and peculiar surface properties [5]. In the field of energetic materials (EM), many researchers are interested in the application of fluorinated graphene in energetic materials because of the F atoms with high chemical activity contained in fluorinated graphene.

Aluminum (Al) and Boron (B) are often added to solid propellant [6,7] as high-energy powder additives due to their high energy density [8,9], but they show some problems, namely easy agglomeration and the reduction of combustion efficiency during the combustion process, respectively. Some progress has been made in promoting the combustion of high-energy powder after adding fluorinated graphene [10,11,12,13,14]. Jiang Yue et al. [11] prepared the aluminum-based composite containing FG and, following molecular dynamics simulations and experiments, proved that FG can significantly improve the combustion performance of aluminum powder due to the existence of the dissociation product-CFx. Wang Jian et al. [14] prepared KNO_3_/B coated with FG. They not only proved that the existence of FG can promote the combustion of boron powder, but also revealed the mechanism for promoting combustion, which involves FG eliminating the inert B_2_O_3_ shell at a low temperature.

High-energy explosives can significantly increase the energy level of solid propellants [15,16,17,18]. Some progress has been made in the field of high-energy explosives after adding fluorinated graphene [19,20]. Tang Pengfei et al. [19] proved, through a quantitative calculation, that a higher degree of fluorination will lead to a lower adsorption energy between CL-20 and FG, and also proved, by testing laser-induced ignition performance, that the laser ignition delay time increased with an increase in the fluorine contents. Zhu Baozhong et al. [20] prepared an ammonium perchlorate (AP)-based composite containing FG and illustrated that FG can reduce the reaction temperature of AP and change the decomposition process of AP.

In this article, in order to understand the characteristics of FG and its influence on the thermal decomposition of nitrate esters, theoretical calculations and experiments were used to study both the thermal stability and thermal oxidation of FG and the effects of FG on the thermal decomposition of absorbent powder (the main ingredient is nitrate esters).

## 2. Method

### 2.1. Computational Details

In this paper, CP2K 8.2 [21] was mainly used for structure optimization and the calculation of ab initio molecular dynamics (AIMD). Based on the structure obtained by CP2K 8.2, and after freezing all atoms, H atoms were used to saturate the boundary for optimization and energy calculation in ORCA 5.0.1 [22,23] in order to obtain the wavefunction for further wavefunction analysis.

**Figure 1:**
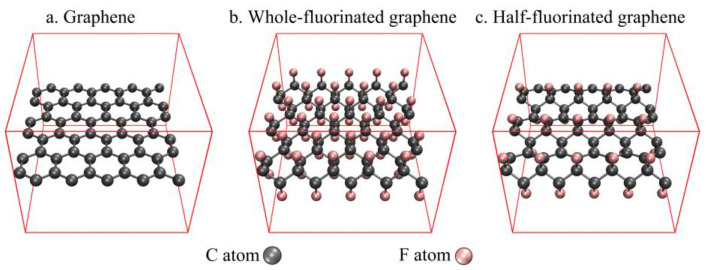
The structures of graphene, whole-fluorinated graphene and half-fluorinated graphene.

**Figure 2:**
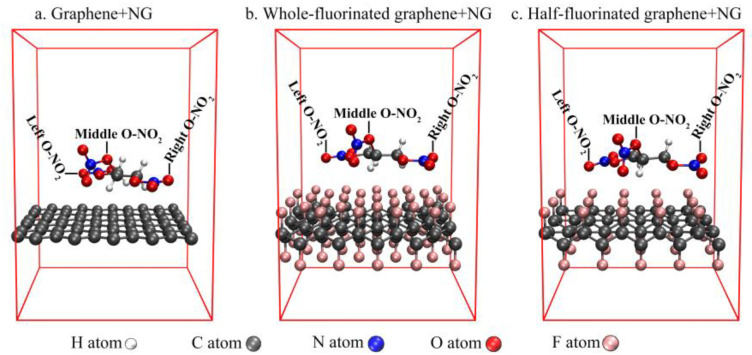
The structure of nitroglycerin (NG) on the surface of graphene, whole-fluorinated graphene and half-fluorinated graphene.

In CP2K 8.2, PBE-D3 functionals [24,25,26] combined with DZVP-MOLOPT-SR-GTH basis set [27,28] were used for structure optimization to obtain the structures of graphene, whole-fluorinated graphene and half-fluorinated graphene (Figure 1). They were also used to optimize the stable structure of nitroglycerin (NG) on the surface of graphene, whole-fluorinated graphene and half-fluorinated graphene (Figure 2).

In order to explore thermal stability, PBE-D3/DZVP-MOLOPT-SR-GTH was used to obtain the 4000 fs AIMD trajectories (step-size is 1 fs) of graphene, whole-fluorinated graphene and half-fluorinated graphene under the CSVR (canonical sampling through velocity rescaling) thermostat and micro-canonical ensemble (NVE). In order to explore the thermal oxidation stability, GFN1-xTB [29] was used to optimize the stable surface model of oxygen molecules on the surface of graphene, whole-fluorinated graphene and half-fluorinated graphene. Then, GFN1-xTB was also used to obtain the 1000 fs AIMD trajectories (step-size is 0.1 fs) of these stable surface models under the CSVR thermostat and micro-canonical ensemble (NVE).

In ORCA 5.0.1, the RIJCOSX technique [30] and the auxiliary basis def2/J [31] were enabled to accelerate all calculations. B3LYP functionals [32] combined with D3BJ dispersion correction [26,33] and the def2-SV(P) basis set [34] was used to optimize the saturation boundary model for nitroglycerin and obtain the wavefunction, wB97M-V functionals [35] combined with the DFT-NL dispersion correction [36,37]. The def2-TZVP basis set [34] was used to calculate the binding energy of the saturation boundary model of nitroglycerin, and the counterpoise correction was simultaneously adapted to solve the basis set superposition error (BSSE) [22,38]. The Multiwfn 3.8 program [39] was used for energy decomposition analysis based on forcefield (EDA-FF) [40] and wavefunction analysis, and molecular structures and isosurface maps were created in the VMD 1.9.3 program [41] based on Multiwfn output files.

### 2.2. Experiment

#### 2.2.1. Materials

Few-layer graphene: its diameter was 0.5–5 μm with a purity of around 99.8wt%. The thickness was 0.8–1.2 nm.

Fluorinated graphene: its diameter was 0.4–5 μm with a purity of 98% The molar ratio of F/C is 29.68–36.63%.

Absorbent powder: this was prepared by mixing uniform and via cooling with centrifugation. 55.5% nitrocellulose, 30.9% nitroglycerin, 12.6% glycerol triacetate and 2% 1.3-Dimethyl-1,3-diphenylurea were its main components.

The composites of absorbent powder and graphene or fluorinated graphene were obtained by mechanical stirring. After being stirred and standing at room temperature for 24 h, the graphene (fluorinated graphene) and powder are mixed in the mass ratio of 2:3.

#### 2.2.2. Instrument

A simultaneous thermal analyzer NETZSCH STA 449 F3 (Selb, Germany) with a platinum furnace was used for TG-DSC analysis. The heating rate was 5 °C/min and the atmosphere was N_2_ or O_2_.

The coupling device NETZSCH STA 449 F3-QMS 403-FTIR (Nicolet iS20, Thermo Fisher Scientific, MA, USA) with a Al_2_O_3_ furnace was used for TG-DSC-MS-FTIR analysis. The heating rate was 5 °C/min and the atmosphere was N_2_.

Thermo Fisher Scientific Nexsa (MA, USA) was used for the testing of X-ray photoelectron spectroscopy (XPS), and the charge correction was carried out with C1s = 284.80 eV as energy standard.

## 3. Results and Discussion

### 3.1. The Properties of Fluorinated Graphene Sample

**Figure 3:**
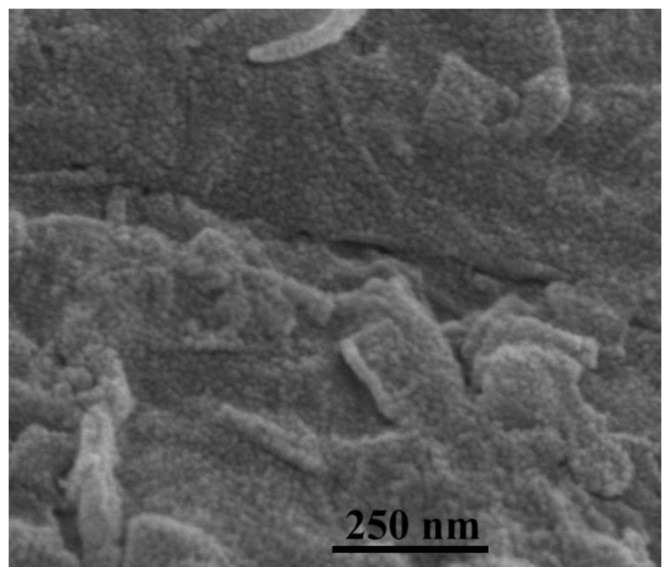
The SEM image of the fluorinated graphene sample.

**Figure 4:**
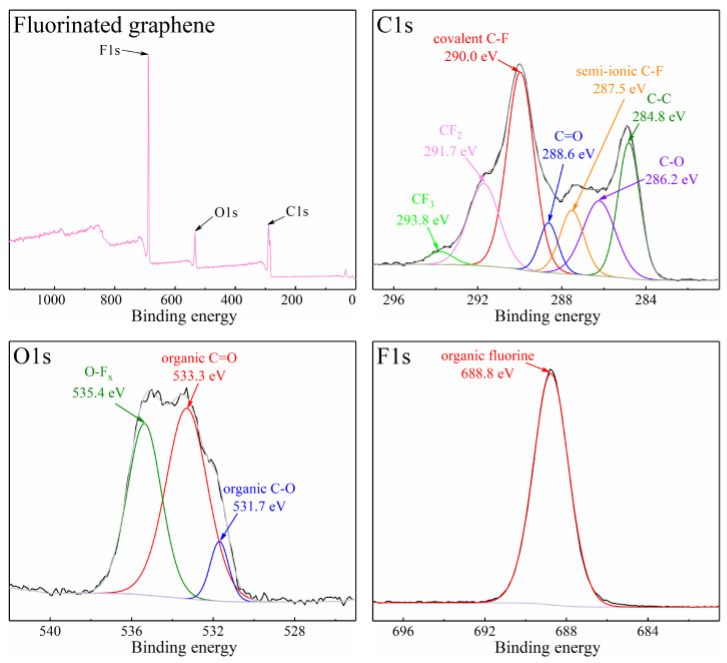
The XPS spectra of C1s, O1s and F1s for the fluorinated graphene sample. The black line is the original data and the grey line is the fitting data.

Figure 3 is the SEM image of fluorinated graphene, and, as can be seen from Figure 3, the surface morphology of fluorinated graphene is relatively uniform. The XPS testing illustrates that the fluorinated graphene sample contains C, F and O, and the atomic concentrations are 68.2%, 21.2% and 10.6%. Figure 4 shows the XPS spectra of C1s, O1s and F1s for the fluorinated graphene sample. Commonly, the binding energy of covalent C-F bond is around 290–291 eV, the semi-ionic C-F bond is around 287–288 eV, and the CF_2_ and CF_3_ groups is around 291–293 eV in the XPS C1s spectrum [42,43]. Therefore, the several peaks in the C1s XPS spectrum correspond to C-C at 284.8 eV, C-O at 286.2 eV, C=O at 288.6 eV, semi-ionic C-F at 287.5 eV, covalent C-F at 290.0 eV, CF_2_ at 291.7 eV, CF_3_ at 293.8 eV, respectively. This matches the results of the O1s XPS spectrum and F1s XPS spectrum in Figure 4. Moreover, the content of C-F bonds, CF_2_ groups and CF_3_ groups in the fluorine graphene sample are 70.5%, 26.2% and 3.3%, respectively.

### 3.2. Thermal Stability and Thermal Oxidation

After fluorinating, π-π packing was destroyed and the layer spacing increased in graphene. Usually, the change in structure will cause a change in thermal stability and thermal oxidation stability. In terms of the application of fluorinated graphene in energetic materials, it is very important to understand the thermal stability and thermal oxidation of FG, but there are few studies on the thermal properties of FG. In this section, the thermal stability and thermal oxidation of graphene and FG are studied via theoretical calculations and experiments.

#### 3.2.1. The Difference in Thermal Stability between Graphene and Fluorinated Graphene

**Figure 5:**
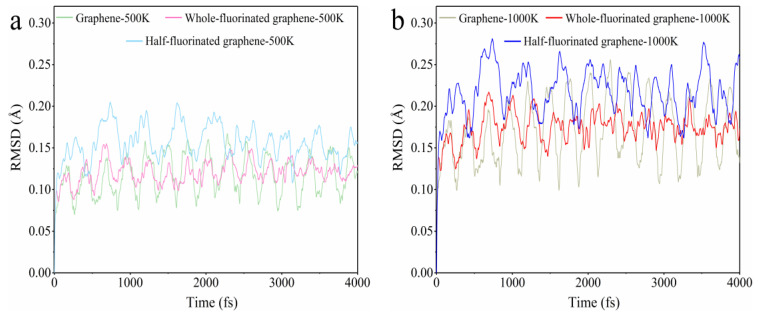
RMSD of AIMD trajectories for graphene, whole-fluorinated graphene and half-fluorinated graphene at 500 K and 1000 K compared with the ground state (0 fs). (**a**) RMSD of AIMD trajectories at 500 K. (**b**) RMSD of AIMD trajectories at 1000 K.

Figure 5 shows the root mean square deviation (RMSD) of AIMD trajectories for graphene, whole-fluorinated graphene and half-fluorinated graphene at 500 K and 1000 K compared with the ground state (0 fs). The larger the RMSD value, the greater the deformation degree [44]. According to Figure 5, all RMSD curves show regular periodic fluctuations. First of all, comparing RMSD curves of the same substance at 500 K and 1000 K, it can be found that the higher the simulation temperature, the greater the geometric fluctuation due to the thermal motion. Secondly, in terms of the maximum values of the RMSD curves for graphene, fluorinated graphene and half-fluorinated graphene, it can be found that half-fluorinated graphene shows the strongest fluctuation and has the worst thermal stability, while graphene and fluorinated graphene have weaker fluctuations and have better thermal stability. Finally, according to the previous discussion, perfect systems (graphene, whole-fluorinated graphene) all show better thermal stability, while imperfect systems (half-fluorinated graphene) show worse thermal stability. Fluorinated graphene produced by current preparation methods [45,46,47] is similar to the imperfect system. Therefore, we speculated that, when compared with graphene, fluorinated graphene has a worse thermal stability in the daily experiment. In the subsequent TG-DSC experiments, our speculation was proved.

**Figure 6:**
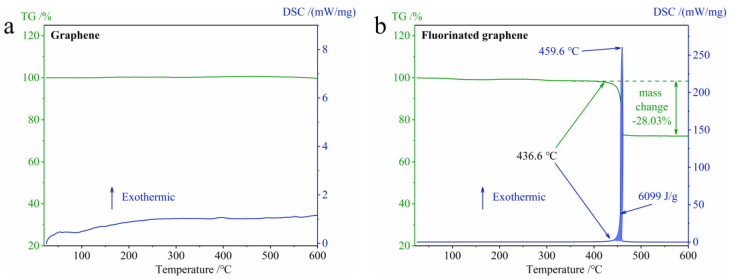
TG-DSC curves of graphene and fluorinated graphene under N_2_ at 25–600 °C. (**a**) TG-DSC curves of graphene. (**b**) TG-DSC curves of fluorinated graphene.

In order to verify our speculation regarding thermal stability, which arose as result of theoretical calculations, the TG-DSC curves of graphene and fluorinated graphene under N_2_ at 25–600 °C were studied, and the results are shown in Figure 6. According to Figure 6, graphene does not decompose up to 600 °C, and fluorinated graphene begins to decompose at around 436.6 °C. Therefore, the thermal stability of fluorinated graphene is worse than that of graphene. This agrees with the discussion relating to thermal stability predicted by the theoretical calculation in Figure 5. In addition, according to Figure 6b, fluorinated graphene decomposes in a short time and emits a large amount of heat, which increases its potential application in the field of energetic materials.

**Figure 7:**
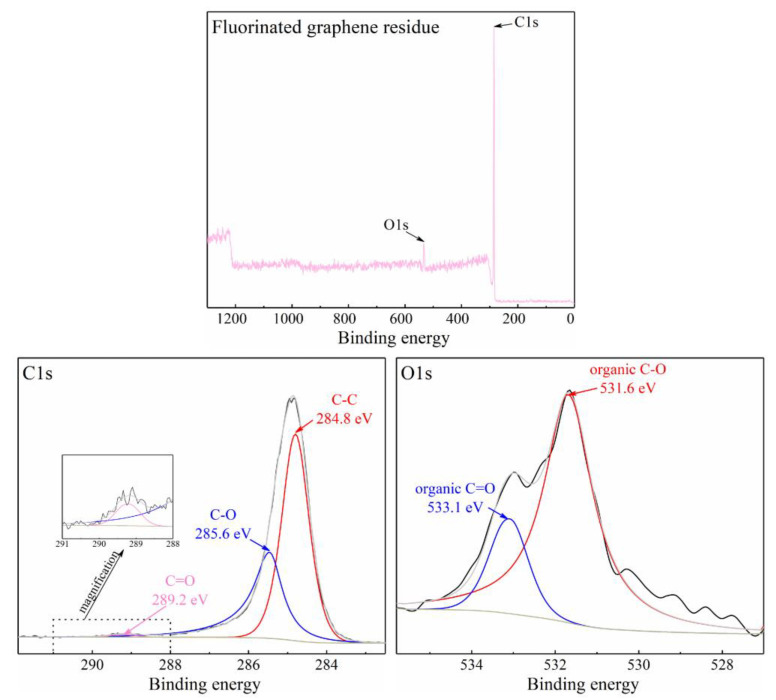
The XPS spectra of the fluorinated graphene residue after TG-DSC experiment. The black line is the original data and the grey line is the fitting data.

Figure 7 shows the XPS result for the fluorinated graphene residue after the TG-DSC experiment. It shows that the main elements in the fluorinated graphene residue are the C element and the O element. There is no obvious peak for the F element in the total spectrum, which indicates that the F elements in the fluorinated graphene sample were completely released as gas during TG-DSC experiment.

#### 3.2.2. The Difference in Thermal Oxidation between Graphene and Fluorinated Graphene

**Figure 8:**
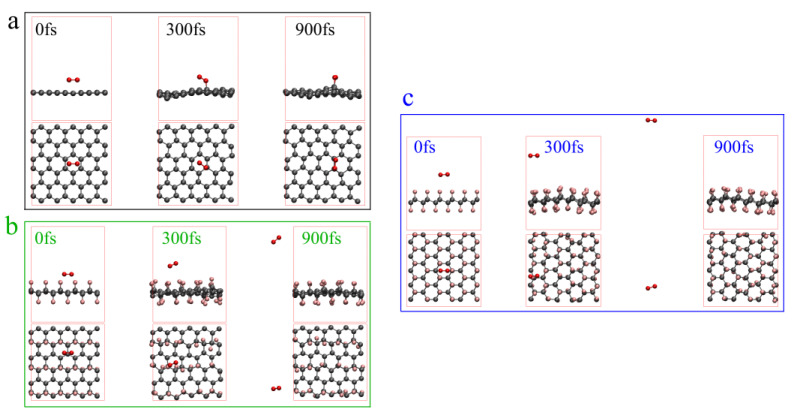
AIMD trajectories at 2000 K with a CSVR thermostat and 0.1 fs step-size. (**a**) Oxygen molecules near the graphene. (**b**) Oxygen molecules near the half-fluorinated graphene. (**c**) Oxygen molecules near the whole-fluorinated graphene.

In order to explore the thermal oxidation of fluorinated graphene, we obtained the AIMD trajectories of oxygen molecules near the graphene, whole-fluorinated graphene and half-fluorinated graphene at 2000 K with a CSVR thermostat. The 0 fs, 300 fs and 900 fs models were selected, whose front and top viewed at 0 fs, 300 fs and 900 fs are shown in Figure 8. According to Figure 8a, oxygen molecules will gradually approach the graphene surface and interact with graphene at 2000 K, which is equivalent to the oxidation process of graphene. According to Figure 8b,c, at 2000 K, oxygen molecules will gradually move away from half-fluorinated graphene and whole-fluorinated graphene over time. This is caused by the large number of electronegative F atoms on their surface. In addition, the more F atoms there are, the farther away the oxygen molecules will be. According to the calculation of AIMD, a clear conclusion can be obtained, and it is that graphene is “aerobic”, whereas half-fluorinated graphene and whole-fluorinated graphene are “anaerobic”. Moreover, the higher the fluorine content, the higher the degree to which the material is “anaerobic”. This has great significance for understanding the thermal oxidation mechanism of fluorinated graphene.

**Figure 9:**
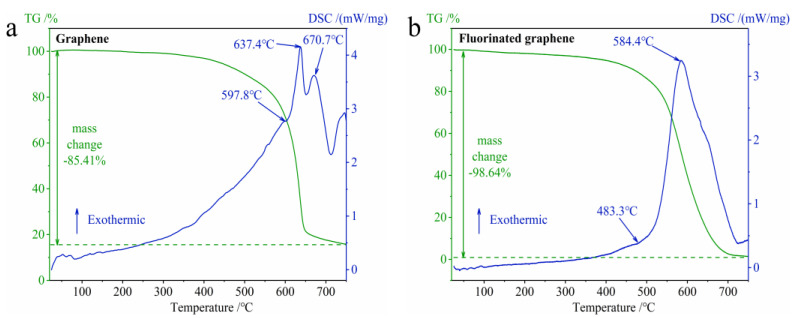
TG-DSC curves of graphene and fluorinated graphene under O_2_ at 25–750 °C. (**a**) TG-DSC curves of graphene. (**b**) TG-DSC curves of fluorinated graphene.

In order to further understand the thermal oxidation properties of fluorinated graphene, the TG-DSC curves of graphene and fluorinated graphene under O_2_ at 25–750 °C were studied, and the results are shown in Figure 9. Figure 9a shows the properties of graphene oxidation. With regard to the TG curve in Figure 9a, the graphene showed a significant mass change in the oxygen atmosphere (the mass begins to decrease significantly at about 400 °C, and the final mass remains about 15%) compared to the nitrogen atmosphere, which was caused by the interaction between oxygen and graphene and can be proved by the previous AIMD results in Figure 8a. The DSC curve in Figure 9a has two exothermic peaks, and the peak temperatures are 637.4 °C and 670.7 °C, respectively. In short, the oxidation of graphene is a direct interaction between graphene and oxygen. Figure 9b shows the properties of fluorinated graphene oxidation. For the TG curve in Figure 9b, the fluorinated graphene thoroughly decomposed in the oxygen atmosphere (the final mass remains about 1.5%) when compared to the nitrogen atmosphere. The DSC curve in Figure 9b has one exothermic peak at 584.4 °C. Considering the previous discussion on the thermal stability of fluorinated graphene and the AIMD results in Figure 8b,c, the thermal oxidation characteristics of fluorinated graphene can be summarized as follows: fluorinated graphene will firstly undergo its own thermal decomposition under the action of heat, and then the thermal decomposition products will further interact with oxygen molecules to achieve the oxidation of fluorinated graphene. This is obviously different from the oxidation properties of graphene.

### 3.3. Theoretical Analysis of the Influence of Graphene or Fluorinated Graphene on Nitroglycerin

#### 3.3.1. The Calculation and Analysis of Binding Energy

The results relating to the binding energy of these structures (NG around the surface of graphene, whole-fluorinated graphene and half-fluorinated graphene) at wB97M-V/def2-TZVP in combination with counterpoise correction can be seen in Table 1.

According to Table 1, the binding energy between NG and series graphene will decrease after graphene is fluorinated, and the lower the fluorine content, the greater the decrease in binding energy. In order to further understand the nature of the binding energy, we conducted energy decomposition analysis based on the AMBER forcefield [48]. The results are shown in Table 2.

First of all, according to Table 2, the difference between the binding energy calculated based on the AMBER forcefield and the binding energy in Table 1 is only about 10 kJ, indicating that the result of energy decomposition based on the AMBER forcefield is credible. Secondly, the dispersion interaction is dominant in “graphene + NG”. This is caused by the π-π stacking between graphene and NG, in addition to the van der Waals interaction, and can be verified by the discussion in Section 3.3.2 below. Finally, in “half-fluorinated graphene + NG” and “whole-fluorinated graphene + NG”, the contribution of electrostatic interaction is much higher than in the case of “graphene + NG”, and the higher the F atoms content, the greater the contribution of electrostatic interaction, which is due to F atoms with extremely high electronegativity.

#### 3.3.2. Interaction Analysis

**Figure 10:**
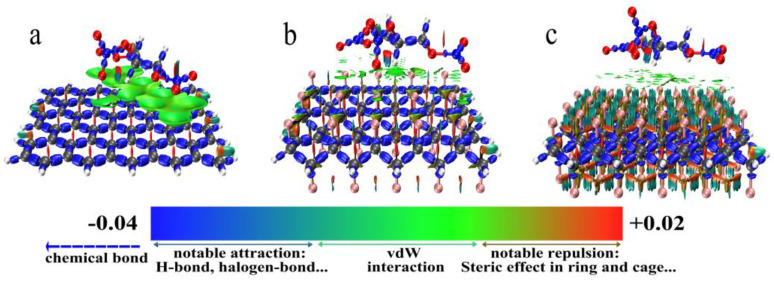
IRI maps with a color scale bar. (**a**) IRI map of graphene + NG. (**b**) IRI map of half-graphene + NG. (**c**) IRI map of whole graphene + NG.

In Figure 10, we plotted the interaction region indicator (IRI) [49] maps for these structures (graphene + NG, half-fluorinated graphene + NG, whole-fluorinated graphene + NG) in order to graphically reveal their interaction regions. According to Figure 10a, it is clear that there is a wide isosurface sandwiched between graphene and NG, which reveals a strong π-π stacking feature. On the contrary, in the Figure 10b or Figure 10c, the isosurface between the fluorinated graphene and NG exhibits intermittent characteristics and only shows the characteristics of van der Waals interaction.

**Figure 11:**
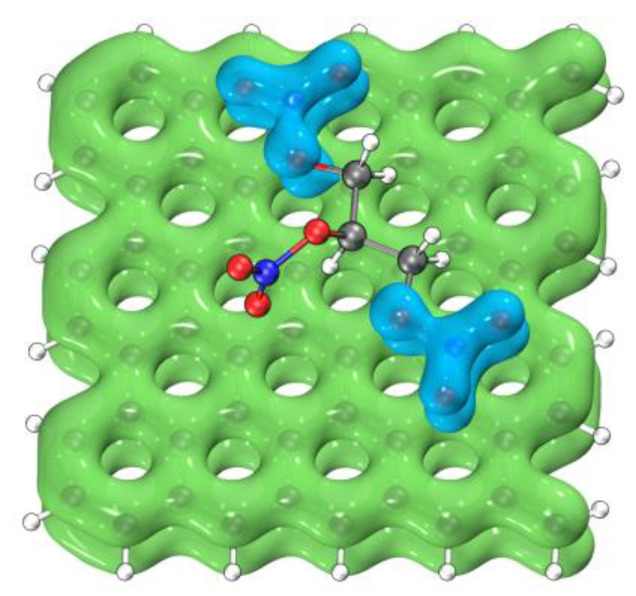
The maps of the isosurface of LOL of some π-LMOs with an isovalue of 0.2. The lime isosurface is the area which belongs to the graphene. The cyan isosurface is the area which belongs to the NG.

Figure 11 shows the area where π-π stacking occurs in the localized molecular orbital (LMO) [50] of graphene + NG. By plotting the isosurface of the localized orbital locator (LOL) [51] of some π-LMOs, it visualizes the interaction between the π orbital of graphene and the π orbital of the nitrate ester group in NG.

#### 3.3.3. The Influence of Fluorinated Graphene on Nitrated Nitrate Group

In this section, the strength of the nitrate groups in NG which belong to different structures (graphene + NG, half-fluorinated graphene + NG and whole-fluorinated graphene + NG) are discussed. The electron density difference between graphene (half-fluorinated graphene and whole-fluorinated graphene) and NG are used to explain the difference in strength.

Commonly, the integration of the electron localization function (ELF) [52] in overlap spaces (ELF-OS) can be used to describe the strength of the bond [53], with a larger ELF-OS value meaning a greater bond strength. Table 3 shows the ELF-OS value of O-NO_2_ in NG which belong to different structures. The positions of O-NO_2_ are shown in Figure 2.

**Figure 12:**
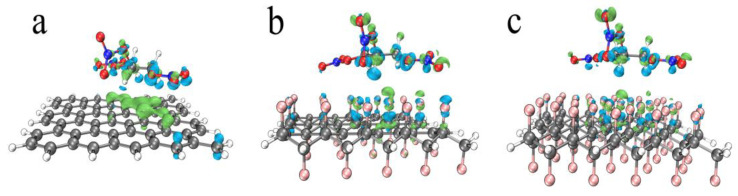
The electron density difference maps. (**a**) Graphene. (**b**) Half-fluorinated graphene. (**c**) Whole-fluorinated graphene.

In Table 3, the values of a single NG are regarded as reference. Through the comparation, the following conclusions can be drawn: First of all, the introduction of graphene, half-fluorinated graphene and whole-fluorinated graphene will slightly change the bond strength of middle O-NO_2_, but it will weaken the bond strength of left O-NO_2_ and right O-NO_2_ in NG, which is defined as the ester weakening effect. Secondly, comparing the magnitude of this effect, graphene shows the strongest ester weakening effect, and half-fluorinated graphene shows the weakest ester weakening effect. Finally, comparing the ester weakening effect of half-fluorinated graphene and whole-fluorinated graphene, it can be explained that the greater the fluorination degree, the stronger the ester weakening effect. In order to further explain the mechanism of the ester weakening effect, electron density difference maps were analyzed. Figure 12 shows the electron density difference maps.

In Figure 12, the isovalue is 0.0007, the lime indicates the main area where the electron density increases, and the cyan indicates the main area where the electron density decreases. It can be seen from Figure 12 that the graphene (half-fluorinated graphene, whole-fluorinated graphene) is where the electron density increases, the O, N atoms of left O-NO_2_ and right O-NO_2_ in NG is where the electron density decreases and the middle area of NG exhibits polarized characteristics. However, it is worth noting that Figure 12a shows extensive electron exchange (the main reason for this is π-π stacking), whereas Figure 12b,c show weak electron exchange. The electron density difference maps explain that electron exchange changes the bond strength of left O-NO_2_ and right O-NO_2_, and the wider the electron exchange, the greater the decrease in bond strength.

In Section 3.3, the effects of graphene and fluorinated graphene on NG are studied via theoretical calculations, and an important conclusion is that the F content in fluorinated graphene results in significant differences in the effect of NG.

### 3.4. The Thermal Decomposition of the Composites

In this section, the thermal decomposition of the composites (graphene + absorbent powder, fluorinated graphene + absorbent powder) is researched for the purpose of understanding the effect of fluorinated graphene on the thermal decomposition of nitrates. TG-DSC and TG-DSC-FTIR-MS are used to achieve this.

**Figure 13:**
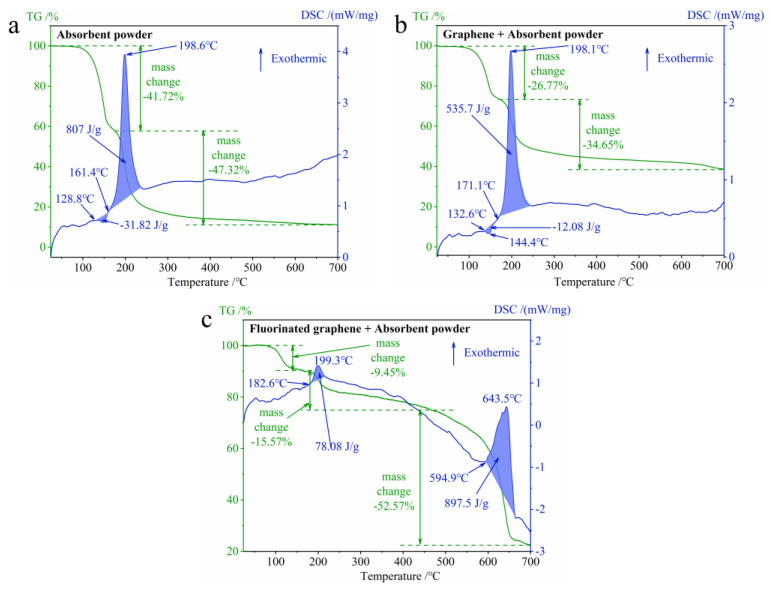
The TG-DSC curves for composites under N_2_ at 25–700 °C. (**a**) Absorbent powder. (**b**) Graphene + absorbent powder. (**c**) Fluorinated graphene + absorbent powder.

Figure 13 presents the results of TG-DSC for composites. First of all, in the Figure 13a, the TG curve for absorbent powder shows two-stage mass loss: 41.72% and 47.32%, respectively. The DSC curve for absorbent powder first shows endotherm with a heat release of −30.82 J/g, which corresponds to the melting of nitrocellulose in the absorbent powder, and it then exhibits exotherm with a heat release of 807 J/g with an exothermic peak temperature of 198.6 °C, which corresponds to the decomposition of energetic substances. Secondly, in Figure 13b, the TG curve for the graphene and absorbent powder composite also shows two-stage mass loss: 26.77% and 36.55%, respectively. This is mainly caused by the introduction of graphene with a better thermal stability. The DSC curve for the graphene and absorbent powder composite also shows endotherm with a heat release of −12.08 J/g at first, and it then shows exotherm with a heat release of 535.7 J/g and an exothermic peak temperature of 198.1 °C. The main reason of the reduction of the heat release is the introduction of graphene, which reduces the ratio of nitrocellulose and the energy level of the composites. Finally, in Figure 13c, the TG curve for the fluorinated graphene and absorbent powder composite shows three-stage mass loss: 9.45%, 15.57% and 52.57%, respectively. The DSC curve for the fluorinated graphene and absorbent powder composite shows two endothermic peaks, with the heat release being 78.08 J/g and 897.5 J/g, respectively, and the exothermic peak temperatures being 199.3 °C and 643.5 °C. It does not show an obvious endothermic peak, which may be caused by the exotherm of the initial decomposition of fluorinated graphene. It is worth noting that the DSC curve in Figure 13c shows two-stage exotherm (199.3 °C, 643.5 °C). Considering Figure 13a (198.6 °C) and Figure 6b (459.6 °C), the first exotherm is related to the decomposition of the absorbent powder under the influence of fluorinated graphene, and the second exotherm is connected to the further reaction of the small molecules from the absorbent powder with the fluorinated graphene. Fluorinated graphene does not affect the decomposition of the powder (the initial exothermic peak temperature is 199.3 °C), but it does greatly increase the heat release of the composites.

**Figure 14:**
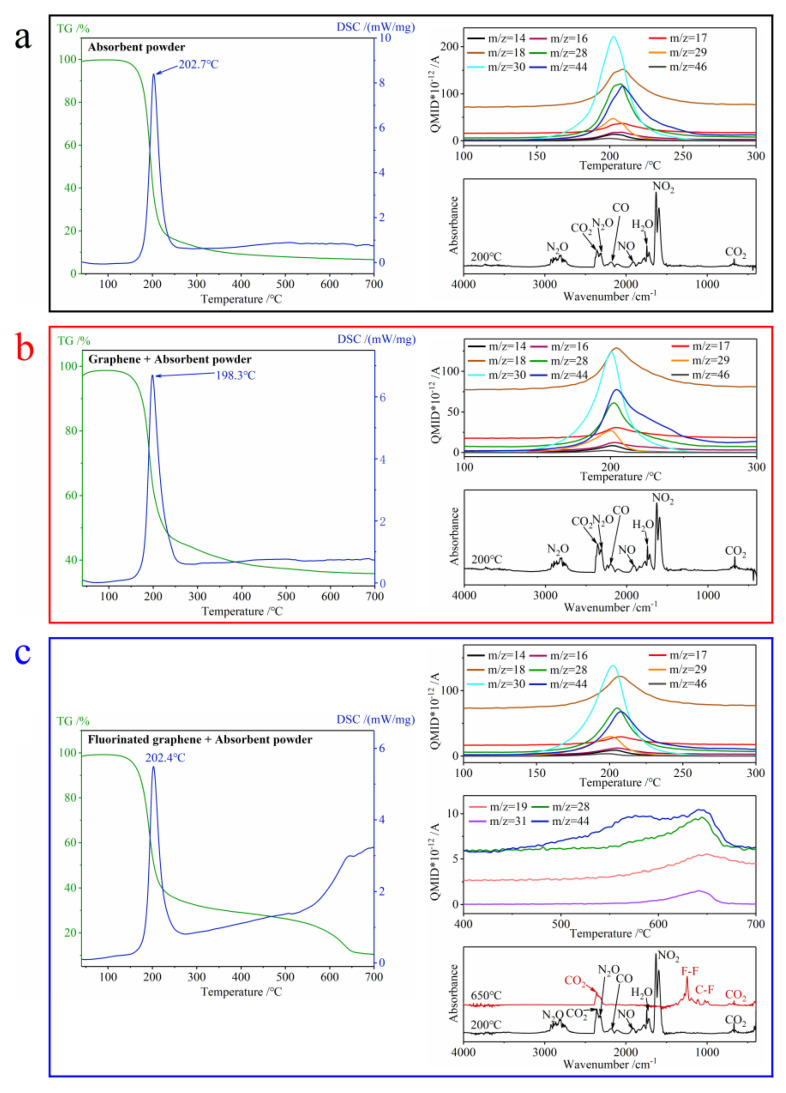
The TG-DSC-MS-FTIR curves of composites under N_2_ at 40–700 °C. (**a**) Absorbent powder. (**b**) Graphene + absorbent powder. (**c**) Fluorinated graphene + absorbent powder.

Figure 14 is the results of TG-DSC-MS-FTIR for composites. In Figure 14a,b, the MS images only show a significant change at around 200 °C, so a FTIR image at 200 °C is given in the lower right of Figure 14a,b respectively. However, for Figure 14c, the MS image also shows changes at around 650 °C besides a significant change at around 200 °C, so FTIR images at 200 °C and 650 °C are given in the lower right of Figure 14c. Firstly, the exothermic peak temperature of the TG-DSC curve in Figure 14 coincides with the peak temperature of the TG-DSC in Figure 13. Secondly, Figure 14a,b show that the composites have the same decomposition products and that these are mainly N_x_O_y_ (x, y = 1, 2), CO and CO_2_. Finally, Figure 14c shows two-stage mass loss, which is the same as in the discussion of Figure 13. The products of the first stage are similar to the products in Figure 14a,b. The vibration peak of the C-F bonds often shows at least two peaks located at 1050–1220 cm^−1^ [43,54]. In Figure 14c, the FTIR image shows peaks at 1000–1300 cm^−1^ when the temperature is 650 °C, and this corresponds to the vibration of C-F bonds produced during the decomposition of the composite (fluorinated graphene + absorbent powder). According to the MS and FTIR images in Figure 14c, the main products during the second stage decomposition are CO_2_ and fluorine-containing substances [55].

## 4. Conclusions

In this paper, both the thermal stability and thermal oxidation of fluorinated graphene and the effect of fluorinated graphene on the thermal decomposition of nitrate esters were studied via theoretical calculations and experiments, and the following four conclusions were obtained.

(1) The thermal stability of fluorinated graphene is poor compared with graphene in daily experiments.
(2) Fluorinated graphene is anaerobic, and the degree of anaerobicity increases as the degree of fluorination increases.
(3) Fluorinated graphene influences the nitrate group in nitrate esters, and the deeper the fluorination degree, the greater this influence is.
(4) Fluorinated graphene greatly increases the heat released during the decomposition of the composites when it is added to the absorbent powder.

In the future, new preparation methods for FG-EM composites and theoretical calculations will greatly promote the application of fluorinated graphene in the field of energetic materials.

## Figures and Tables

**Table 1:** The binding energy at wB97M-V/def2-TZVP combined with counterpoise correction.

	Graphene + NG	Half-Fluorinated Graphene + NG	Whole-Fluorinated Graphene + NG
Binding energy	−93.73	−59.09	−70.54

**Table 2:** The results of the energy decomposition analysis based on the AMBER forcefield.

	Graphene + NG	Half-Fluorinated Graphene + NG	Whole-Fluorinated Graphene + NG
Electrostatic	−1.31	−7.07	−8.56
Dispersion	−162.12	−95.85	−112.09
Repulsion	69.67	35.41	38.43
Total	−93.77	−67.52	−82.22

**Table 3:** ELF-OS value of O-NO_2_ in different NG.

	NG	Graphene + NG	Half-Fluorinated Graphene + NG	Whole-Fluorinated Graphene + NG
Left O-NO_2_	1.05	0.95	0.99	0.97
Middle O-NO_2_	0.98	0.98	0.99	0.98
Right O-NO_2_	1.03	0.95	0.98	0.97

## Data Availability

Data are contained within the article.
